# Recurrent suicide attempts affect normalization of HPA axis dysregulation after recovery from major depression

**DOI:** 10.3389/fpsyt.2022.937582

**Published:** 2022-08-12

**Authors:** Johannes M. Hennings, Marcus Ising, Manfred Uhr, Florian Holsboer, Susanne Lucae

**Affiliations:** ^1^Translational Department, Max Planck Institute of Psychiatry, Munich, Germany; ^2^Department of Dialectical Behavioral Therapy, kbo-Isar-Amper-Klinikum Munich-East, Munich, Germany; ^3^HMNC Brain Health GmbH, Munich, Germany

**Keywords:** suicidality, depression, DEX/CRH (dexamethasone/corticotropin releasing hormone) test, HPA, suicide attempt

## Abstract

More than 700,000 people worldwide die by suicide every year, and the number of suicide attempts is estimated as 20 times higher, most of them being associated with psychiatric disorders, especially major depression. Knowledge about effective methods for preventing suicide attempts in individuals at high risk for suicide is still scarce. Dysregulation of the neuroendocrine stress response system, *i.e*., the hypothalamic-pituitary-adrenocortical (HPA) axis, is one of the most consistent neurobiological findings in both major depression and suicidality. While the HPA axis is mostly overactive in depression, individuals with a history of suicide attempts exhibit an attenuated hormonal response to stress. It is unknown, however, whether the HPA axis is constantly attenuated in repeated suicide attempters or whether it regains normal responsivity after recovery from depression. Using the combined dexamethasone suppression/corticotropin-releasing hormone (dex/CRH) test, we assessed HPA axis regulation in acute depression (*N* = 237) and after recovery with respect to previous suicide attempts. Patients without previous suicide attempts show normalization of the stress hormone response to the second dex/CRH (basal ACTH response and cortisol response) after recovery from acute depression, while patients with multiple previous SA show an increased ACTH response. The change in HPA axis responsivity in patients with only one previous SA lies between the response patterns of the other groups with no change in HPA axis reactivity. Our findings suggest that patients with a history of suicide attempts belong to a subgroup of individuals that exhibit a distinct pattern of stress hormone response during acute depression and after recovery. Future studies may extend our approach by investigating additional psychological stress tasks to gain a broader understanding of the stress pathology of recurrent suicide attempters.

## Introduction

While patients repeatedly engaging in suicidal behavior often feel left unsupported, healthcare professionals struggle themselves in order to address their needs (1, 2) and do not know how to prevent suicide attempts efficaciously in the future (3). More than 700,000 people worldwide die by suicide every year, and the number of suicide attempts (SA) is estimated as 20 times higher (4). Across psychiatric disorders, risk factors for suicidal behavior such as childhood maltreatment, non-suicidal self-injury, and previous SA have been identified (5, 6) but the predictive ability has not improved over the years, according to a recent meta-analysis (7).

Up to 70% of suicides have been linked to affective disorders (8), which in turn are associated with dysfunctional stress coping and, in particular, dysregulation of the neuroendocrine stress response system, *i.e*., the hypothalamic-pituitary-adrenocortical (HPA) axis (9). Dysregulation of the HPA axis turned out to be one of the most predictive biomarker-associated suicide risks (10, 11). Interestingly, while overactivity of the HPA axis is a common finding, especially in severe depression (12), an attenuated stress hormone response has been observed in individuals with suicidal behavior (13–17). It is unclear how these findings fit together with HPA axis hyperactivity documented in severely depressed patients with high suicidality. Hypercortisolism predicted suicide and suicidal behavior in major depression in some studies using the dexamethasone-suppression test (DST) (10, 18, 19). With respect to its sensitivity and specificity, however, the DST has been questioned (20). Further and conflictingly, more recent studies using a psychological stress paradigm showed an attenuated cortisol response in patients with a history of suicide attempts or with an increased risk for suicide (14, 16). With respect to these controversial findings, the combined dexamethasone suppression/corticotropin-releasing hormone stimulation (dex/CRH) test may provide some advantages for the assessment of the HPA axis in suicidality as it (1) has been recommended for the assessment of the HPA axis in affective disorders due to higher sensitivity and specificity (20, 21) and (2) comprises, besides the DST, a direct CRH stimulation component that would presumably be more comparable to the stress paradigms applied in more recent studies.

The stress-diathesis model of suicidality (22, 23) indicates multiple factors, such as psychopathology, genetics, early life experiences, social interactions and stress, physical illness, and neurobiology, to determine one's predisposition to suicidal behavior. From a psychological point of view and according to the interpersonal theory of suicide (24), suicide risk is mainly driven by the feeling of being a burden on others, thwarted belongingness, and a learned ability to hurt oneself, on the one hand. On the other hand, suicidal behavior can be regarded as an acquired dysfunctional technique of problem-solving (25), and subjects that experienced inescapable situations in youth may have “learned” to react with suicidal behavior, including suicidal thoughts as a possible emotional escape from an otherwise unescapable situation. In fact, in patients with a history of SA, it has been observed, that ideating, preparing, or attempting suicide under conditions that are perceived as highly aversive (such as despair or guilt in affective disorders) may give hope for an escape from the condition and reduce mental pain (25–27). This clinical observation has been substantiated by functional neuroimaging studies showing altered activity and connectivity of specific brain regions in suicide attempters (28, 29) and during suicide ideation (30), including regions that are functionally connected to the HPA axis such as the pre-frontal cortex [reviewed in (28)]. Remarkably and counterintuitively at a first glance with respect to HPA axis overactivity observed in severe depression (9), previous studies showed that the HPA axis of patients ideating or having attempted suicide responds with an attenuated stress hormone response upon a stress paradigm (14–16), suggesting that these individuals belong to a subgroup with distinct HPA reactivity to mental stress. Interestingly in this regard, an attenuating effect of previous SA on the HPA axis can be found also during acute depression (31), and this effect increases with the number of previous SA.

Reduced basal cortisol in patients with a previous SA has been interpreted as a ‘burnt-out' HPA axis after a long period of chronic stress in the fore-front of the suicide attempt (32) and blunted stress hormone response has been coined a marker of suicide risk (14, 15). Nevertheless, the function and pathophysiology of an attenuated HPA axis associated with suicidality are not yet understood. It is further unknown whether the attenuation of the HPA axis is transient, indicating a distinct hormonal response to mental stress (such as the calming effect of suicidality in some individuals), or whether it is a sustained effect in terms of a vulnerability marker. We, therefore, assessed the HPA axis response with the combined Dex/CRH test during a state of mental stress, i.e., an (at least moderately severe) acute depressive episode, and a second time after recovery (*N* = 237) hypothesizing that previous suicide attempts may impact on the normalization of HPA axis reactivity.

## Methods

### Sample description

In this study, 237 Caucasian in patients suffering from at least moderately severe depressive episode (54.9% females, mean age 47.9 ± 13.3 (SD) years) participating in the Munich Antidepressant Response Signature (MARS) project (33) were included. Diagnoses were obtained by trained psychiatrists in accordance with the diagnostic criteria of the Diagnostic and Statistical Manual of Mental Diseases (DSM-IV). The exclusion criteria were depressive syndromes secondary to any medical or neurological condition (e.g., intoxication, drug abuse, and stroke), the presence of manic, hypomanic, or mixed affective symptoms, a lifetime diagnosis of alcohol dependence, illicit drug abuse, or the presence of severe medical conditions (e.g., ischemic heart disease). As described in detail previously (33), patients were included within 5 days after admission to the clinic for the treatment of an acute depressive episode (Table 1). An overview of study procedures is depicted in the Supplementary Figure.

**Table 1:** Sample characteristics.

	**All** ^a^		**No previous SA**		**1 previous SA**		**>1 previous SA**			
	**(*****N*** **= 237)**		**(*****N*** **= 181)**		**(*****N*** **= 43)**		**(*****N*** **= 13)**		***P*** ^b^	***post***−***hoc***^c^
Age, mean (SD), y	47.9	(13.3)	48.8	(13.4)	44.1	(12.5)	48.5	(12.4)	0.146	
Female gender, No (%)	130	(54.9%)	90	(49.7%)	31	(72.1%)	9	(69.2%)	**0.017**	noSA < prevSA
Actual suicide attempt, No (%)	24	(10.1%)	1	(0.6%)	19	(44.2%)	4	(30.8%)	**<0.001**	noSA < prevSA, mprevSA
Previous suicide attempt, No (%)										
2-3, No (%)							8	(61.5%)		
>3, No (%)							5	(38.5%)		
Diagnosis										
Bipolar depression, No (%)	21	(9.1%)	21	(11.7%)	1	(2.4%)	1	(8.3%)	0.126	
Single MDE, No (%)	77	(33.5%)	60	(33.3%)	16	(38.1%)	1	(8.3%)		
Recurrent depression, No (%)	132	(57.4%)	99	(55.0%)	25	(59.5%)	10	(83.3%)		
Age at disease onset, mean (SD), y	36.9	(14.8)	38.8	(14.9)	31.4	(13.5)	29.1	(11.4)	**0.002**	prevSA < noSA
Previous depressive episode, mean (SD)	47.9	(13.3)	48.8	(13.4)	44.1	(12.5)	48.5	(12.2)	0.081	
HAM-D at admission, mean (SD)	26.5	(6.8)	26.0	(6.7)	28.4	(6.6)	27.2	(7.4)	0.103	
HAM-D at discharge, mean (SD)	7.3	(5.3)	6.9	(5.2)	8.4	(4.9)	9.4	(6.3)	**0.046**	ns
Early partial response, No (%)	160	(69.9%)	124	(70.5%)	29	(72.5%)	7	(53.8%)	0.418	
Response at discharge, No (%)	190	(86.4%)	149	(87.6%)	32	(86.5%)	9	(69.2%)	0.176	
Remission at discharge, No (%)	159	(72.3%)	129	(75.9%)	23	(62.2%)	7	(53.8%)	0.074	
Treatment resistance at admission, No (%)	36	(16.4%)	30	(18.0%)	2	(5.0%)	4	(30.8%)	**0.048**	prevSA < mprevSA
Duration of hospital stay, mean (SD), weeks	11.0	(6.7)	9.6	(6.4)	11.2	(8.3)	10.7	(6.8)	0.289	
Employment										
employed, No (%)	162	(71.7%)	125	(72.7%)	29	(70.7%)	8	(61.5%)	0.537	
unemployed, No (%)	21	(9.3%)	13	(7.6%)	6	(14.6%)	2	(15.4%)		
retired/invalidity allowance, No (%)	43	(19.0%)	34	(19.8%)	6	(14.6%)	3	(23.1%)		
Living with family/partner, No (%)	137	(60.6%)	110	(64.0%)	21	(51.2%)	6	(46.2%)	0.177	
Current alcohol abuse, No (%)	19	(8.2%)	10	(5.6%)	8	(19.0%)	1	(7.7%)	**0.017**	noSA < prevSA
Current benzodiazepine abuse, No (%)	26	(11.3%)	19	(10.7%)	6	(14.6%)	1	(8.3%)	0.735	

a *For some variables, there are missing data and N does not equal the number of total patients. Percentages are based on available data*.

b *Kruskal-Wallis and Chi-square, respectively*.

c *Significant post-hoc comparisons with Bonferroni correction*.

*Bold values indicate significant contrasts*.

The study was approved by the local Ethics Committee of the Medical Faculty at Ludwig Maximilians University, Munich, Germany and was carried out in accordance with the latest version of the Declaration of Helsinki.

### Diagnosis and psychopathology assessment

Diagnoses were obtained by trained psychiatrists according to the criteria of the Diagnostic and Statistical Manual of Mental Diseases (DSM-IV). Diagnoses were confirmed with the modified version of the Munich-Composite International Diagnostic Interview (DIA-X/M-CIDI) (34). Psychopathology was assessed weekly during hospital stay by continuously trained raters using the 21-item Hamilton Depression Rating Scale (HAM-D) (35). Patients with at least moderately severe depression (HAM-D≥14) entered the analysis. Pharmacological treatment was assessed from patients' charts, and antidepressant dosages were adjusted according to the specific therapeutic plasma level range. Early partial response was defined as an at least 25% HAM-D reduction after 2 weeks, response as an at least 50% reduction after 5 weeks compared to the HAM-D at admission, and remission at discharge as a HAM-D <10. Antidepressant treatment resistance at admission was defined as when patients have failed to respond to at least two trials with different antidepressants given in adequate dosages for at least 6–8 weeks. Continuously trained raters assessed suicidality on admission using the categories of item 3 in the HAM-D rating scale, classifying patients as “no suicidality” (score 0), “weary of life” (score 1, “Feels life is not worth living”), “suicide ideations” (score 2, “Wishes he/she were dead or any thoughts of possible death to self”, or 3, “Ideas or gesture of suicide”), and “suicide attempt” (scoring 4 at item 3). Actual (leading to the current admission) and previous suicide attempts were further assessed in the clinical interview (17).

### Combined dex/CRH test

Hypothalamic-pituitary-adrenocortical axis regulation was analyzed within a few days after admission (mean 6.93 days +/– 2.93 SD) to the hospital and at discharge using the combined dexamethasone suppression/corticotropin-releasing hormone (dex/CRH) test. The dex/CRH test was performed as described in detail previously (20). As previous data suggested a psychopathology-independent influence of medication with carbamazepine and lithium on plasma cortisol and ACTH levels (12), patients receiving these drugs were not included in this study. Briefly, subjects were pretreated with 1.5 mg of dexamethasone per os at 11 p.m. At 2.30 p.m. the following day, a venous catheter was placed, and blood was drawn into tubes containing EDTA and Trasylol (Bayer, Leverkusen, Germany) at 3 p.m., 3.30 p.m., 3.45 p.m., 4 p.m., and 4.15 p.m. An intravenous bolus of 100 μg of human CRH (Ferring, Kiel, Germany) was given at 3.02 p.m. A radioimmunoassay kit was used for the measurement of plasma cortisol concentrations (CT Cortisol RIA, DRG Diagnostics, Marburg, Germany). Plasma ACTH concentrations were assessed by an immunoradiometric assay (cobas ECLIA, Roche Diagnostics, Rotkreuz, Switzerland).

For the analysis of hormonal tests, we calculated the area under the concentration curve (AUC) for ACTH (*A*_AUC_) and cortisol (*C*_AUC_) using trapezoidal integration. Besides the AUC, we also assessed the basal ACTH and cortisol (i.e., after dexamethasone but at 3 p.m., immediately before CRH, *A*_bas_, *C*_bas_).

### Statistical analysis

Due to inhomogeneous group sizes and a significant Kolmogorov-Smirnov test of normality deviation (*P* < 0.001 for all hormonal parameters), non-parametric statistics were used for all analyses. Demographic data, baseline clinical data, and treatment outcome variables were compared using the Pearson χ^2^ test in the case of qualitative data and with the Kruskal-Wallis test for independent samples in the case of quantitative data. Pre-post comparisons were performed using the Wilcoxon test for paired samples. Spearman's rank coefficients were calculated for correlation analyses. *P* < 0.05 was set to be significant. In the case of multiple comparisons in *post hoc* analyses, Bonferroni-corrected *P*-values are reported. Statistical analyses were performed using SPSS (version 27.0.1.0, IBM Cooperation, USA).

## Results

### Clinical characteristics

Of 237 patients (54.9% females) included in this study, 181 (76.4%) did not have any previous suicide attempt, 43 (18.1%) had 1 previous suicide attempt, and 13 (5.5%) reported more than one previous suicide attempt. The rate of women was higher in patients with a previous suicide attempt compared to patients without previous suicide attempts (Pearson χ^2^; *P* = 0.017). Prior to admission, 24 (10.1 %) attempted suicide; among them all, except one, reported previous suicide attempts, too (Pearson χ^2^ test; *P* < 0.001; refer to Table 1 for details). Age at disease onset was significantly lower in patients with one previous suicide attempt compared to patients without previous suicide attempts (31.4 vs. 38.8 years; Kruskal-Wallis with *post hoc* Bonferroni correction; *P* = 0.011). The rate of treatment-resistant patients was higher in the group of patients with multiple suicide attempts compared with patients with one suicide attempt (30.8% vs. 5.0%; Pearson χ^2^ test; *P* < 0.048). Alcohol abuse was lower in patients without previous suicide attempts compared to patients with one previous attempt (5.6% vs. 19.0%; Pearson χ^2^ test; *P* < 0.017). There was no significant difference between groups with respect to age, diagnosis (bipolar depression, single MDE, and recurrent depression), HAM-D at admission and discharge, early partial response, response or remission status at discharge, duration of hospital stay, employment status, living with family/partner, or current benzodiazepine abuse (Table 1). There was also no difference between groups regarding antidepressant medication (refer to Supplementary Table 1) that could have influenced HPA measurements (36). Although HAM-D decreased in all three groups (Table 1) and most patients had any suicidal symptomatology at discharge (94.7, 89.2, and 76.9%, respectively), a small number of patients still suffered from weariness of life (4.1%, 8.1%, and 15.4%, respectively) or even suicidal ideations (1.2% (*N* = 2), 2.7% (*N* = 1), and 7.7% (*N* = 1), respectively), while differences across groups were not significant (Pearson χ^2^ test; *P* < 0.147).

### Effects of previous suicide attempts on HPA axis response to the dex/CRH test

There was no significant difference between patients without, with one, or multiple previous suicide attempts neither at admission for basal ACTH (*A*_bas_) (7.9 ±7.0 pg/ml vs. 6.8 ± 4.3 pg/ml vs. 5.2 ± 2.1 pg/ml; *H*(2) = 2.62; *P* = 0.270), ACTH response (*A*_AUC_) (1,282.2 ± 996.6 pg/ml vs. 1,103.0 ± 1,099.6 pg/ml vs. 769.2 ± 305.3 pg/ml; *H*(2) = 5.03; *P* = 0.081), basal cortisol (*C*_bas_) (22.0 ± 27,9 ng/ml vs. 19.7 ± 21.8 ng/ml vs. 14.6 ± 6.1 ng/ml; *H*(2) = 0.146; *P* = 0.929), and cortisol response (*C*_AUC_) (3,492.6 ±3,298.9 ng/ml vs. 2,641.7 ±2,892.7 ng/ml vs. 2,324.2 ± 2,269.3 ng/ml; *H*(2) = 3.963; *P* = 0.138).

### Change between admission and discharge responses to the dex/CRH-test

**Figure 1:**
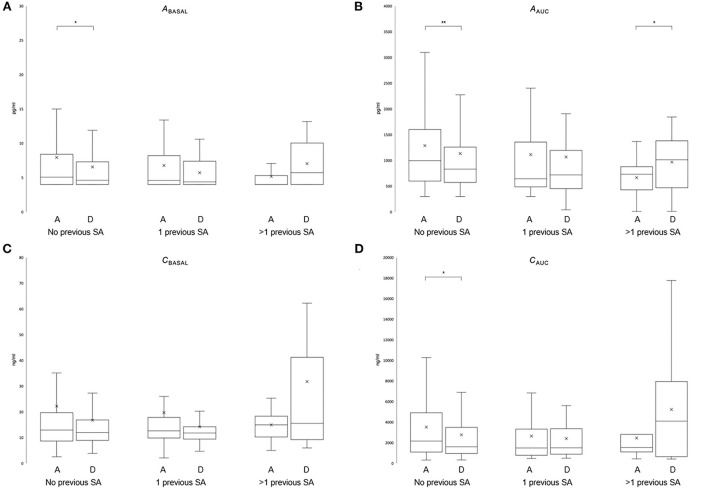
Effect of previous suicide attempts on the HPA axis during recovery of depression. Stress hormone levels in the dex/CRH test are depicted [A_bas_ **(A)**; A_AUC_ **(B)**; C_basal_ **(C)**; C_AUC_ **(D)**] at hospital admission **(A)** and discharge **(D)** in patients without previous SA (*N* = 181), with one previous SA (*N* = 43) or more than one previous SA (*N* = 13). The Wilcoxon test for paired samples (see text for details). The whisker lines correspond to highest and lowest values no further than the 1.5 interquartile range from the hinges. Median lines are indicated across the boxes, and mean values are indicated with an × (*P < 0.05; **P < 0.01).

Comparing the change in hormonal levels between admission and discharge in patients without a suicide attempt revealed a significant decrease in basal ACTH and ACTH and cortisol responses; *A*_bas_ (−1.5 ± 7.0 pg/ml *Z* = −2.122; *P* = 0.034), *A*_AUC_ (−166.7 ± 973.4 pg/ml; *Z* = −2.606; *P* = 0.009), and *C*_AUC_ (−795.5 ± 3,232.3 ng/ml; *Z* = −2.559; *P* = 0.011). The change in *C*_bas_ (−5.8 ± 29.0 ng/ml; *Z* = −1.929; *P* = 0.054) was not significant. While no significant changes were observed in patients with one suicide attempt (*A*_bas_: −1.0 ±0.4 pg/ml *Z* = −1.664; *P* = 0.096, *A*_AUC_: 4.9 ± 708.1 pg/ml; *Z* = −0.175; *P* = 0.861, *C*_bas_: −5.4 ± 18.7 ng/ml; *Z* = −1.457; *P* = 0.145, and *C*_AUC_: −169.5 ± 2,588.9 ng/ml; *Z* = −0.519; *P* = 0.604), patients with multiple SA showed a significant increase in *A*_AUC_ (572.3 ± 787.4 pg/ml; *Z* = −2.191; *P* = 0.028). The increases in *A*_bas_ (2.1 ± 4.4 pg/ml *Z* = −1.244; *P* = 0.214), *C*_bas_ (3.8 ± 12.9 ng/ml; *Z* = −0.459; *P* = 0.646), and *C*_AUC_ (1,843.9 ± 2,987.4 ng/ml; *Z* = −1.478; *P* = 0.139) were not statistically significant (Figure 1). Due to the high variance in hormonal parameters, we added an analysis of log (ln)-transformed values, yielding the same results (Supplementary Paragraph 1).

A SA prior to admission occurred at different times in the two SA groups, which might have had an impact on the HPA axis irrespective of the number of previous SAs; therefore, we reran the analysis excluding patients with SA prior to admission (*N* = 237–24 = 213). We found a similar pattern of hormonal changes: In patients without previous SA, both basal and stress hormone responses decreased (*A*_bas_: −1.5 ± 7.1 pg/ml, *Z* = −2.179, *P* = 0.029; *A*_AUC_: −170.6 ± 983.2 pg/ml; *Z* = −2.635; *P* = 0.008; *C*_basal_: −5.9 ± 29.3 ng/ml, *Z* = −1.975, *P* = 0.048; and *C*_AUC_: −809.7 ± 3,267.0 ng/ml; *Z* = −2.525; *P* = 0.012) between admission and discharge. In multiple SA, there was again a significant increase in ACTH (*A*_AUC_: 572.3 ± 787.4 pg/ml; *Z* = −2.191; *P* = 0.028), while patients with single SA showed no significant changes.

## Discussion

This study investigated for the first time the temporal dynamics of HPA axis reactivity during recovery from acute depression with respect to previous suicide attempts. Overall, while neither the severity of depressive symptomatology at admission nor remission rates at discharge were different between groups, patients with previous SA suffer from a higher burden of disease as documented by an earlier disease onset, a higher rate of treatment resistance in the current episode, and a higher rate of alcohol abuse. Although there were no significant differences in basal or hormonal response levels between groups at admission, patients with recurrent and without previous SA showed an opposing dynamic with respect to HPA axis reactivity in the combined dex/CRH test during recovery from depression. While patients without previous suicide attempts show a reduction in stress hormone response in the dex/CRH (*A*_bas_, *A*_AUC_, and *C*_AUC_; when excluding recent SA also for *C*_bas_) between admission and discharge, patients with multiple previous SA show an increase in stimulated ACTH (*A*_AUC_). The HPA axis reactivity of patients with only one previous SA did not change, and thus, ranges between the other two groups.

These findings are remarkable in several aspects: First, hyperactivity of the HPA axis is a common finding in acutely depressed patients (9), and normalization of the hormonal excess chaperons successful antidepressant treatment (33, 37). Thus, at a first look, it is hard to explain why some patients (i.e., multiple suicide attempters), although not different in depression severity and ability to recover compared to others (i.e., patients without previous suicide attempts), show an increase in HPA axis reactivity during recovery. Non-normalization of the HPA axis (here in terms of reduction in hyperactivity) after antidepressant treatment has been associated with early relapse of depression (38–40) and increased suicide risk (10). As previous SA is a risk factor for both (18, 19, 41, 42), an increase in HPA reactivity in previous suicide attempters at discharge as observed in this study may explain some of these earlier findings. Thus, non-normalization or an increase in the HPA axis during depression treatment may be a biological characteristic for individuals with a history of SA.

Second, it is still unclear whether HPA axis reactivity is only acutely altered in suicide attempters or resembles a stable vulnerability marker. In a prospective study, attenuated stress hormone response after a psychosocial stress task predicted later SA (13). The reduced cortisol response to stress has also been found in the offspring of parents with a history of SA (15) or completed suicide (14), indicating that sustained or phasic hypocortisolism preceded a suicide attempt, and hair cortisol was reduced in suicide attempters (43). In addition, several studies showed reduced baseline cortisol in patients with SI or a previous SA (16, 44, 45). Plausibly, hyporeactivity of the HPA axis was regarded as a risk marker of SA, indicating impaired activation of the HPA axis in response to stress. Nevertheless, in a previous analysis, we could not replicate a negative correlation of HPA response with previous SA at hospital admission after recovery from depression at discharge, arguing against the hypothesis of an attenuated HPA axis as a trait marker (31). There are no further studies using repeated HPA assessment during a depressive episode with respect to previous SA. Thus, the increase in stimulated ACTH in patients with multiple SA after recovery in this study may be the expression of a certain “normalization” or regained reactivity of the HPA axis in these patients.

Third, our findings may add to the understanding of the HPA axis in suicidality. It has been speculated that cortisol non-suppression in the dexamethasone suppression test (DST) as observed in former studies is (1) either associated with completed suicides or (2) primarily associated with non-response to antidepressant treatment and only secondary to increased suicide risk (11, 42). In a large depression sample, recent suicide attempters showed a reduced HPA response in the dex/CRH test (17), and previous suicide attempts were negatively correlated with CRH-induced ACTH and cortisol levels independent of depression severity (31). While not reaching significance, we observed a similar direction of effect in this study at the admission dex/CRH test. In addition, several studies using psychological test tasks in individuals with previous suicide attempts show consistently a hyporeactive HPA axis compared to controls (14–16). Thus, taking all these observations together, antecedents of previous SA and recent SA attenuate HPA reactivity, an effect that can also be observed during acute depression (in comparison to patients without previous SA). Although being still reactive, HPA activity seems to be inverted in the latter: in situations of mental stress (including major depression), previous suicide attempters exhibit an attenuation of the HPA axis (14–17). O'Connor and co-workers (16), for example, showed that participants who had made a previous suicide attempt exhibited a significantly lower cortisol response to a multidimensional stress test compared to suicide ideators and controls. Interestingly, in their study, subjects who attempted within the past year showed a blunted cortisol response compared to subjects with a more distant history of SA. Adding the observation of an increased stress hormone response after recovery from depression made in this study, it can be speculated that the attenuation of the HPA axis during mental stress in these particular subjects may be transient. Further studies, including repeated assessments of the HPA axis, are needed to prove this hypothesis.

Nevertheless, the meaning of an altered HPA axis in SA remains still obscure and interpretations of the findings can only be vague. While the increase of ACTH in patients with multiple SA observed here argues against the hypothesis of a “burned-out,” blunted HPA axis due to repeated stress (32), the assumption of a psychologically calming effect of SA and SI has been repeatedly documented in individuals engaging in suicidal behavior (25, 26, 46). The latter has led to the hypothesis of reinforced suicidality as ideating or attempting suicide can opt a way out of situations that are experienced as unbearable, and though suicidality can become a learned behavior, causing relief from mental pain (25, 47). Thus, a transient attenuation of stress hormones appears plausible from this point of view, and alterations of the HPA axis in recurrent SA may be a biological marker of this mechanism.

While cortisol measurements during a psychological stress task let speculations about the level of regulation widely open, higher cerebral instances of stress hormone regulation are bypassed in the dex/CRH test by the direct administration of CRH. Thus, alterations in stress hormone regulation in previous suicide attempters might be downstream to the hypothalamus. Interestingly, in this context, epigenetic mechanisms have been implicated in HPA axis hypoactivity in SA-mediated, possibly *via* GR downregulation and enhanced GR sensitivity (43). Sustained stress transmitted by a depressive episode, in contrast, translates into cortisol receptor resistance (9), an effect that may have superimposed the independent impact of suicidality on cortisol in our study.

The results of our study still need to be interpreted cautiously due to the high variability of hormonal levels and inhomogeneous group sizes. Furthermore, given a small sample size in the group of patients with multiple SA, power may have been insufficient to detect significant effects. In a previous analysis, we found small effects of nicotine consumption on ACTH but not cortisol, whereas caffeine intake, BMI, and the number of attempts to place the venous catheter did not influence ACTH or cortisol response in the dex/CRH test (12). Nevertheless, compared to basal hormonal measurements or the DST, the dex/CRH test is advantageous with respect to sensitivity and robustness (21), and patients receiving carbamazepine or lithium were excluded from analysis due to their known psychopathology-independent influence on the HPA axis (12, 48). Sex-specific analyses revealed that the effect of decreasing stress hormones (*A*_bas_ and *A*_AUC;_ data not shown) in patients without previous SA is mainly produced by men, while the hormonal increase during recovery occurs in women with multiple SA (*A*_AUC_ and *C*_AUC_; data not shown). While gender has a well-known impact on the HPA axis, the observed differences may also be related to a female majority in the group of patients with multiple SA. Notwithstanding, this is the first observation of an inverted dynamic of HPA axis reactivity during acute and recovery of depression with respect to previous SA that needs replication in independent samples. To further elucidate the pathophysiology of different forms of mental stress in individuals with a history of SA, it would be appropriate to apply psychological stress tasks in addition to the dex/CRH test during acute depression and after recovery. The direct comparisons of HPA axis reactivity in the dex/CRH test with psychological stress tasks may allow further insights into the anatomical and molecular level of altered HPA axis regulation.

## Conclusion

This is the first study analyzing the dynamics of HPA axis reactivity during acute depression and recovery with respect to previous SA. We show that patients without previous SA show a reduction in stress hormone response in the dex/CRH during recovery from acute depression, while patients with multiple previous SA show an increase in stimulated ACTH, suggesting that patients with a history of suicide attempts belong to a subgroup of individuals that exhibit a distinct pattern of stress hormone response to states of mental stress (such as major depression). Future studies may replicate these initial findings, extended by an additional psychological stress task and a thorough assessment of suicidal psychopathology, hypothetically also promising to detect new options for anti-suicidal interventions and to define patient subgroups that benefit from individual treatment.

## Data availability statement

The datasets presented in this article are not readily available because the data has not been approved for public dissemination by the Ethics Committee. Requests to access the datasets should be directed to johannes.hennings@kbo.de.

## Ethics statement

The studies involving human participants were reviewed and approved by LMU Munich. The patients/participants provided their written informed consent to participate in this study.

## Author contributions

JH, MI, MU, FH, and SL: conceptualization, methodology, and writing—review and editing. JH: data curation, writing—original draft preparation, and visualization. JH, MI, MU, and SL: investigation. MI, FH, and SL: supervision and validation. FH: resources. All authors contributed to the article and approved the submitted version.

## Funding

This study was supported in part by a research grant from the German Federal Ministry of Education and Research (BMBF, FKZ 01ES0811).

## Conflict of interest

The authors declare that the research was conducted in the absence of any commercial or financial relationships that could be construed as a potential conflict of interest.

## Publisher's note

All claims expressed in this article are solely those of the authors and do not necessarily represent those of their affiliated organizations, or those of the publisher, the editors and the reviewers. Any product that may be evaluated in this article, or claim that may be made by its manufacturer, is not guaranteed or endorsed by the publisher.

## Abbreviations

ACTH: adrenocorticotropic hormone
*A*_AUC_: ACTH AUC
AUC: area under the concentration curve
BMI: body mass index
*C*_AUC_: cortisol AUC
CRH: corticotropin-releasing hormone
dex/CRH test: combined dexamethasone suppression/corticotropin-releasing hormone test
GR: glucocorticoid receptor
HAM-D: 21-item Hamilton depression rating scale
HPA axis: hypothalamus-pituitary-adrenocortical axis
MAOI: monoamine oxidase inhibitor
MARS: Munich Antidepressant Response Signature
MDE: major depressive episode
NaSSA, noradrenergic: specific serotonergic antidepressant
OR: odds ratio
SD: standard deviation
SNRI: serotonergic-noradrenergic reuptake inhibitor
SSRI: selective serotonin reuptake inhibitor
TCA: tricyclic antidepressant.
