# Supplementary material for: Recurrent suicide attempts affect normalization of HPA axis dysregulation after recovery from major depression
Source: Front Psychiatry. 2022 Aug 12;13:937582. doi: 10.3389/fpsyt.2022.937582 (PMC9412752; doi:10.3389/fpsyt.2022.937582)
Supplement: Supplementary file 1 [file Data_Sheet_1.PDF]

Recurrent suicide attempts affect normalization of HPA axis dysregulation after recovery from major depression

Running title: Recurrent suicide attempts and normalization of HPA axis

Supplemental material:

1 supplemental paragraph

1 supplemental figure

1 supplemental table

Supplemental Paragraph 1

Effects of previous suicide attempts on HPA axis response to the dex/CRH test

Using log (10)-transformed hormonal parameters, there was no significant difference between patients without, with one or multiple previous suicide attempts neither at admission for basal ACTH (A_{bas}), ACTH response (A_{AUC}), basal cortisol (C_{bas}) and cortisol response (C_{AUC}) (P=.270, P=.081, P=.929 and P=.138, respectively) nor at discharge (P=.370, P=.175, P=.716 and P=.553, respectively).

Change between admission and discharge responses to the dex/CRH-test

Log (10)-transformed hormonal parameters comparing hormonal levels between admission and discharge in patients without a suicide attempt revealed a significant decrease of basal ACTH and ACTH and cortisol responses (A_{bas}: Z=-2.222; P=.026), A_{AUC}: Z=-2.460; P=.014, and C_{AUC}: Z=-

2.812; P=.005). The change in C_{bas} (Z=-1.664; P=.096) was not significant. While no significant changes were observed in patients with one suicide attempt (A_{bas}: Z=-1.460; P=.144, A_{AUC}: Z=-.058; P=.953, C_{bas}: Z=-1.194; P=.232, and C_{AUC}: Z=-.882; P=.378), patients with multiple SA showed a significant increase of A_{AUC} (Z=-1.988; P=.47). The increases of A_{bas} (Z=-1.362; P=.173), C_{bas} (Z=-.357; P=.721) and C_{AUC} (Z=-.866; P=.386) were not significant.

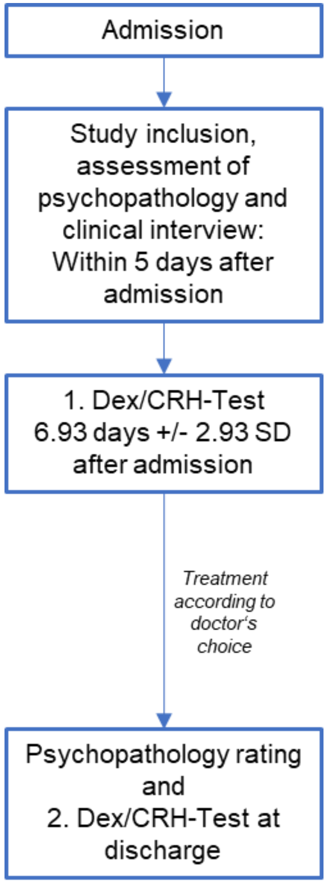

Supplemental Figure 1: Study protocol and procedures.

Supplemental Table 1. Medication at admission dex/CRH

	All[a]		no SA		1 prev SA		>1 prev SA		*P*[b]
SSRI	*65*	27.4%	44	24.3%	16	37.2%	5	38.5%	.154
SNRI	*37*	20.7%	37	20.4%	10	23.3%	2	15.4%	.818
TCA	*33*	13.9%	27	14.9%	6	14.0%	0	0.0%	.324
NaSSA	*55*	23.2%	44	24.3%	7	16.3%	4	30.8%	.428
NARI	*5*	2.1%	5	2.8%	0	0.0%	0	0.0%	.454
Mood stabilizers[a]	*41*	17.3%	34	18.8%	6	14.0%	1	7.7%	.483
Antipsychotics	*38*	16.0%	27	14.9%	9	20.9%	2	15.4%	.626
Benzodiazepines	*90*	35.2%	65	35.9%	19	44.2%	6	46.2%	.496

SSRI, selective serotonin reuptake inhibitor; SNRI, serotonergic-noradrenergic reuptake inhibitor; TCA, tricyclic antidepressant; NaSSA, noradrenergic, specific serotonergic antidepressant; NARI, noradrenaline reuptake inhibitor.

[a] Mood stabilizers excluding carbamazepine and lithium.

[b] *P*-values from Pearsons Chi-Square test.
